# Examination of program exposure across intervention delivery modes: face-to-face versus internet

**DOI:** 10.1186/1479-5868-4-7

**Published:** 2007-03-12

**Authors:** Rebekah M Steele, W Kerry Mummery, Trudy Dwyer

**Affiliations:** 1 School of Health and Human Performance, Central Queensland University, Rockhampton Queensland, Australia; 2 School of Nursing and Health Studies, Central Queensland University, Rockhampton Queensland, Australia

## Abstract

**Background:**

There has been increasing interest in the ability of the internet to produce behaviour change. The focus of this study was to describe program exposure across three intervention groups from a randomised trial (RT) comparing traditional face-to-face, internet-mediated (combined internet plus face-to-face), and internet-only program delivery.

**Methods:**

Baseline and immediately post-intervention survey data, and exposure rates from participants that commenced the RT were included (n = 192). Exposure was defined as either face-to-face attendance, website usage, or a combination of both for the internet-mediated group. Characteristics of participants who were exposed to at least 75% of the program material were explored. Descriptive analysis and logistical regression were used to examine differences between groups for program exposure.

**Results:**

All groups showed decrease in program exposure over time. Differences were also observed (χ^2 ^= 10.37, p < 0.05), between intervention groups. The internet-mediated (OR = 2.4, 95% CI 1.13–5.1) and internet-only (OR = 2.96, 95% CI 1.38–6.3) groups were more likely to have been exposed to at least 75% of the program compared to the face-to-face group. Participants with high physical activity self-efficacy were 1.82 (95% CI 1.15–2.88) times more likely to have been exposed to 75% of the program, and those allocated to the face-to-face group were less likely to have attended 75% of the face-to-face sessions if they were classified as obese (OR = 0.21 95% CI 0.04–0.96).

**Conclusion:**

These results suggest that the internet groups were as effective as the face-to-face delivery mode in engaging participants in the program material. However, different delivery methods may be more useful to different sub-populations. It is important to explore which target groups that internet-based programs are best suited, in order to increase their impact.

## Background

Current epidemiological evidence suggests that approximately 60% of the global population do not participate in sufficient physical activity for health benefit [1], defined as participating in 30-minutes of moderate-intensity physical activity (of least 10-minute bouts) on 5 or more days of the week [2]. Inactivity is associated with many chronic diseases including cardiovascular disease, type 2 diabetes, coronary heart disease and some cancers [3-7]. Therefore, developing effective physical activity programs that are widely accessible is a necessity, given the rising rates of sedentary living and associated health implications.

Group-based programs that offer face-to-face treatments are common and popular strategies in behaviour change research [8]. Unfortunately, face-to-face programs often have high running costs, limited access and availability, and high attrition rates [9-12]. Drop out rates have been reported as high as 50% within 6-months of beginning a program [13], which further limits the public health impact of face-to-face programs. Recently, researchers have focused upon innovative interactive health communications (IHC) such as the internet and computer expert systems, as a means of engaging populations and individuals in health-related behaviour change [14-17]. Studies in this area suggest that IHC mediums, particularly the internet, can offer an alternative method or mediated approach to intervention delivery. Reported advantages of the internet include; widespread access, tailoring of information, online social support, instantaneous interactivity, and confidentiality and anonymity [10].

In physical activity research only a small number of internet-based trials have been reported [16-19]. A study by Marshall and associates (2003) showed that a stage-based physical activity website significantly decreased sitting time, and increased motivational readiness for physical activity. They also reported that only 26% of participants logged on to the website more than once, and a later publication [20], reported that of the 327 participants randomised to the website condition, only 46% accessed the website a minimum of once. A meta-analysis also highlighted the difficulties associated with user engagement and retention in internet-based interventions [21]; which can result in high drop-out rates and reduced intervention exposure. Examining strategies to enhance program exposure is important, however we also need to explore participant characteristics and identify target groups that may be more responsive to internet-based program compared to traditional face-to-face delivery.

We recently reported the results of a randomised trial (RT) comparing intervention delivery modes for a 12-week physical activity intervention (Health-*e*Steps) [22]. The RT compared traditional face-to-face delivery (FACE) with a combined face-to-face plus internet delivery (internet-mediated [IM]), and an internet-only (IO) group. Results of the RT showed all groups significantly increased in self-reported mean minutes of activity; there were no differences between intervention groups; and all groups were statistically equivalent from baseline to the post-intervention follow-up [22]. The Health-*e*Steps intervention was based upon social cognitive theory (SCT) [23] and self-management [24], and aimed to provide users with the skills required to adopt and maintain an active lifestyle. We aimed to build upon previous research, and enhance website retention and engagement by (i) conducting a rigorous formative evaluation process, (ii) including additional face-to-face contact (Internet-Mediated group), (iii) ensuring a dynamic and changing website as opposed to a static information-only site, (iv) including up-to-date local community information and, (v) providing individualised and personally relevant feedback. Additionally, one of the unique aspects of the website was that the modules were delivered on a week-by-week basis; the entire website could not be accessed in one sitting, a disadvantage noted by others [18,19].

The focus of this study was to; (a) describe program exposure [website usage and face-to-face attendance] across the three delivery modes of the Health-*e*Steps RT, (b) examine the association of program exposure and change in physical activity behaviour and, (c) identify predictors of program exposure. Specifically, we examined characteristics of participants who were exposed to at least 75% of the program (face-to-face attendance or website usage). Participant exposure has been found to be a strong influencing factor on the success of program outcomes [25] including internet-based interventions [16].

## Methods

### Participants and intervention

The recruitment, randomisation and intervention protocol have been previously reported [22]. However, briefly participants were recruited via local media advertising, and randomly allocated into one of the three intervention groups (n = 192), eligibility included (i) ≥ 18-years; (ii) functionally mobile ≥ 10 minutes; (iii) inactive; (iv) access to the internet; and (v) signed informed consent. The Health-*e*Steps intervention [26] included a variety of modules/activities focusing upon; lifestyle activity, benefits and barriers, goal setting, self-monitoring, resistance training, self-talk, self-reinforcement, time and stress management, relapse prevention, and social support. Each week focused upon a different module topic and included weekly activities combining self-management skills, (problem solving, decision making, resource utilisation, action planning and self-tailoring) with SCT constructs [26]. For example, teaching the self-management skills decision making and problem solving, enables people to identify their own concerns and problems, and act within a logical sequence to overcome the situation. Therefore enabling individuals to understand relapse situations, understand why we respond negatively to certain situations, and thus be able to identify and implement techniques to prevent relapse (e.g. activity cues, positive self-talk).

Intervention participants allocated to the FACE group received weekly 1-hour face-to-face contact sessions with a trained program facilitator (degree qualified in health education/health promotion). The IM group received the same content delivered via the internet, and also received two additional face-to-face sessions, facilitated by the same facilitator as the FACE group. The IO group received access to the same program content as the IM group via the internet. Participants were also given the opportunity to receive incentives (pedometer for 'walking buddy', water bottles, socks, two $50 gift voucher), via email and website access for the IM and IO groups, and face-to-face attendance for the FACE group. The incentives required participants to write a short sentence in response to a physical activity related question. To be able to enter the draw the FACE participants were required to be 'in attendance' at a face-to-face session. The IM and IO group were required to logon to the website to view the question and post their response back via an email link in the website. For example, in Week 2 pedometers were offered to participants who responded with a short sentence on '*Who is your walking buddy or social support person? *and, '*why would a pedometer be useful to them?*' Incentives were offered in Weeks 2, 4, 6, 8 and 10.

Access to an online Health-*e*Steps representative/support person, Nutritionist (support for the nutrition information included in one of the weekly modules) and Exercise Physiologist (support for the stretching and resistance training exercises included in one of the weekly modules) were also provided to participants via an email link in the internet-groups. FACE participants had the opportunity to interact with the facilitator and guest presenter. The RT was approved by the Central Queensland Human Research Ethics Committee, all participants provided written informed consent.

### Outcome measures

Measurements for this study were collected at baseline (Week 1) and immediately post-intervention (Week 12). Questionnaires were self-report and self-administered. All groups had face-to-face contact for data collection.

#### Demographic

Demographic variables included age, gender, occupation and employment status. Height and weight measures were calculated using a calibrated stadiometer and digital scale. Body mass index (BMI) was calculated as weight [kg]/height [m]^2^. History of internet use was recorded based upon length of experience (6 months, 6–12 months, 1–1.5 months, > 2 years, > 3 years). Internet self-efficacy (ISE) was also assessed [27]. Eight questions relating to hardware and software technologies/capabilities (confidence to surf the web; use of chat-rooms, email, download information; use of search engines [google/yahoo] and browsers [Internet Explorer/Netscape], and confidence with hardware such as networks and servers), were included based upon a five point Likert-type scale ranging from 'not confident at all' to 'very confident'. The ISE questionnaire is scored by dividing the sum of all items by the number of items [27].

#### Physical activity

Self-report physical activity was assessed using the Active Australia survey. The Active Australia survey asks questions related to moderate- and-vigorous intensity activities performed for a period of at least 10-minutes [28]. Test-retest reliability of the Active Australia has been previously established, and the instrument has been reported to have satisfactory convergent validity with previously established survey tools used in Australia [29,30]. The scoring protocol has been previously reported [22,28].

#### Physical Activity Self-efficacy (PASE)

Physical activity self-efficacy (PASE) was assessed using a five-point Likert-type scale questionnaire, which asked people to rate their confidence in performing physical activity in a range of conditions and situations from 'not confident at all' to 'very confident' (e.g. 'I am confident I can be physically active when there is no one to be active with', 'I am confident I can be active when it is very hot outside'). This questionnaire has been used in previous studies [31,32]. Cronbach's alpha for the PASE Questionnaire items has been reported as 0.76 [31]. It is scored by summing the responses and dividing by the total number of items.

#### Social Support for Physical Activity (SSPA)

Social support for physical activity (SSPA) was assessed using a five-point Likert-type scale questionnaire. Participants were asked four items related to how often ('never' to 'very often') over the last three months their family, friends and/or colleagues have supported them to be physically active (e.g. 'During the past three months how often have people done something to help you be physically active?', 'During the last three months how often have people done or offered to do physical activity with you?'). This questionnaire has previously been reported to have a Cronbach's alpha of 0.77 [31], and is scored by summing the responses and dividing by the total number of items.

#### Program exposure

Exposure was tracked using weekly attendance rolls in the FACE group, which were collated by the facilitator. A combination of attendance rolls (collated by the same facilitator) and *web tracking *was used to track exposure across the IM group. Exposure in the IO group was tracked using the same *web tracking *system used for the IO group. Specifically, *web tracking *for the IM and IO groups was collected using Advanced Web Statistics 5.9 (AWStats) [33]. AWStats is a web server logfile analyser that provides user statistics including visit's, unique visitors, pages, hits, rush hours, browsers, broken links, and HTTP errors.

Drop-outs were defined as not completing follow-up data in Week 12 or previously stating that they wanted to withdraw from the study. Seventy-five percent *program exposure *was operationally defined as attending or being *exposed *to a minimum of 75% of the module/information sessions whether they were delivered face-to-face or via the internet. In this context, participants who achieved 75% program exposure, attended and/or logged onto the website a minimum of 7.5 times over the intervention period (excluding Weeks 1 and Week 12 as they involved face-to-face data collection). Dunn, Garcia et al. [34] report that exposure to at least 66.6% of program sessions is an adequate intervention dose, for this study the exposure rate was increased to 75% to reflect a more conservative approach. The definition of *program exposure *for the intervention groups was based upon the assumption that participants that logged on to the Health-*e*Steps website actually read and interacted with the website material. This is similar to the assumption that those who attend face-to-face sessions actually listen and engage with the program facilitator/program material.

### Analysis

#### Weekly program exposure

Descriptive statistics of participant characteristics and weekly program exposure were analysed and reported using the mean (± SD), or numbers and percentages.

#### Program exposure and change in physical activity

Seventy-five percent program exposure was dichotomised into yes (achieved 75% program exposure) and no (didn't achieve 75% program exposure). Chi-square analysis was first used to examine differences in 75% program exposure by intervention group. Analysis of Covariance (ANCOVA) was used to examine the effect of *at least 75% program exposure *and change in the primary outcome measure (mean minutes of physical activity participation), controlling for age, gender, BMI, as well as baseline ISE, PASE, SSPA, and group allocation. Physical activity was logarithmically transformed owing to its skewed distribution, and analysis conducted on the transformed variable.

#### Predictors of program exposure

Logistic regression was used to examine participant characteristics and likelihood of achieving at least 75% program exposure. Seven variables (group allocation, gender, age, BMI, ISE, PASE and SSPA) representing baseline characteristics were used. First, regression analysis was used to examine predictors of program exposure by demographic variables (gender, age and BMI, ISE and, group allocation) for the entire cohort. Second, we included PASE and SSPA as psychosocial variables in the model, given their strong association with regular physical activity participation [35]. Analyses were also conducted stratified by group. SPSS Version 12 was used for all analysis and significance accepted at p < 0.05.

## Results

### Baseline characteristics

The intervention attracted predominately women (83.3%) with an overall mean age of 38.4 years (± 11.2) and BMI of 32.2 (± 7.5) (Table 1). At baseline differences in PASE and ISE were found between the FACE and IM groups (p < 0.05). No other differences were observed. Overall 80% (52/65) of the FACE, 78.5% (51/65) of the IM, and 90.3% (56/62) of the IO groups completed the 12-week follow-up.

**Table 1:** Descriptive Summary of Participant Baseline Characteristics: Overall Study Population, Face-to-Face Group, Internet-Mediated Group, and Internet-Only Group

**Variable**	**INTERVENTION GROUP**			
	**Total** (n = 192)	**FACE** (n = 65)	**IM** (n = 65)	**IO** (n = 62)
**Gender **No. (*%*)				
Male	32 (16.7%)	7 (10.8%)	14 (21.5%)	11 (17.7%)
Female	160 (83.3%)	58 (89.2%)	51 (78.5%)	51 (82.3%)
**Age (y) **Mean (*SD*)	38.7 (12.0)	37.6 (12.4)	39 (13.0)	39.6 (10.5)
**Body Mass Index **Mean (*SD*)	32.1 (7.53)	31.3 (7.63)	32.0 (7.53)	32.0 (7.74)
**Employment Status **No. (*%*)				
Full-time	108 (56.3%)	36 (55.4%)	37 (56.9%)	35 (56.5%)
Part-time	38 (19.8%)	12 (18.5%)	13 (20%)	13 (21%)
Home Duties	16 (8.3%)	5 (7.7%)	4 (6.2%)	7 (11.3%)
Student	17 (8.9%)	5 (7.7%)	9 (13.8%)	3 (4.8%)
Not working/Retired	13 (6.8%)	7 (10.8%)	2 (3.1%)	4 (6.5%)
**History of Internet Use **No. (*%*)				
< 6-months	23 (12%)	11 (16.9%)	4 (6.2%)	8 (12.9%)
6 – 12 months	9 (4.7%)	2 (3.1%)	4 (6.2%)	3 (4.8%)
1 – 1.5 years	14 (7.3%)	7 (10.8%)	4 (6.2%)	3 (4.8%)
> 2 years	23 (12%)	7 (10.8%)	9 (13.8%)	7 (11.3%)
> 3 years	123 (64.1%)	38 (58.5%)	44 (67.7%)	41 (66.1%)
**Internet Self-efficacy# **Mean (*SD*)	3.36 (0.88)	3.16 (0.9)	3.55 (0.74)*	3.37 (0.96)
**Physical Activity Self-efficacy**, Mean (*SD*)	2.98 (0.71)	2.84 (0.70)	3.18 (0.75)*	2.92 (0.64)
**Social Support for Physical Activity**, Mean (*SD*)	2.36 (0.66)	2.20 (0.70)	2.39 (0.66)	2.49 (0.61)
**Activity Status **No. (*%*)				
Inactive	157 (81.8%)	52 (80%)	53 (81.5%)	52 (83.9%)
Active	35 (18.2%)	13 (20%)	12 (18.5%)	10 (16.1%)
**Physical Activity **(minutes/week) median (*25–75 percentile*)	47.5 (0–108.8)	60.0 (0–107.5)	40.0 (0–105.0)	42.5 (0–120)
**At least 75% Program Exposure **No. (*%*)	43.8% (93)	27.7% (18)	50.8% (33)	53.2% (33)
**Drop-out **No. (*%*)	17.2% (33)	20% (13)	21.5% (14)	9.7% (6)

*Note*. FACE = face-to-face; IM = internet-mediated; IO = internet-only *p < 0.05 *Post Hoc *IM> FACE; # Five Point Likert Scale

### Weekly program exposure

**Figure 1:**
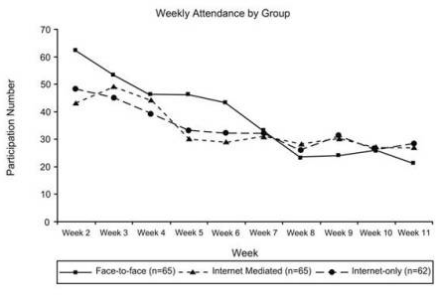
**Weekly Exposure during the intervention period for the Face-to-Face Group, Internet-Mediated Group, and Internet-Only Group**. Face-to-Face = number of participants at each weekly face-to-face session; IM = number of participants that logged onto the website at least once each week and attended face-to-face sessions in Weeks 5 and 9; IO = number of participants that logged onto the website at least once each week. Weeks 1 & 12 are excluded due to face-to-face data collection.

The mean number of face-to-face sessions (exposure) attended by the FACE group was 6.1 (excluding Week 1 & 12 due to data collection, and drop-outs). The mean number of logins (exposure) for the IM group was 11.5 and 11.8 for the IO group. However, internet access ranged from 2–102 times for the IM group, and 2–90 times for the IO group over the entire intervention period. Taking into account multiple logins each week for the internet-groups, overall average attendance for all three groups was 5.5 sessions (SD ± 2.3). A decline in weekly exposure was observed for each intervention group (Figure 1).

Participants were also given the opportunity to receive incentives. Although not specifically examined, observation showed only small numbers of participants engaging in the opportunity to receive incentives. For example, we found 72% (91/127) of participants from the IM, and IO groups logged on to the website in Week 2, in which there were a number of incentives available. IM and IO groups received email notification of the incentives via the weekly email reminders, and the website homepage also highlighted the incentive opportunities. However, of the 91 participants who logged on to the site during Week 2, only 21 (20%) participants responded to the 'incentive opportunity'. Participants in the IM and IO groups were also given the chance to receive online advice and support from a Health-*e*Steps representative, exercise physiologist, and a registered nutritionist via email. However, use of these email contacts was minimal. No emails were received by the Exercise physiologist, only 4 were received by the Nutritionist, and 20 were received by the Health-*e*Steps representative. The majority of emails received by the Health-*e*Steps representative were for the resetting of forgotten passwords.

### Program exposure and change in physical activity

Overall, approximately 44% of participants demonstrated at least 75% program exposure, 61% achieved 60% program exposure, and 70% were exposed to at least half of the program material. Significant differences in program exposure was found for those exposed to at least 75% of the program material by intervention group (χ^2 ^= 10.4, p < 0.05), with the FACE group showing the lowest with-in group program exposure rate of 28%, followed by 51% and 53% for the IM and IO groups respectively (Table 1). ANCOVA showed that 75% program exposure significantly influenced mean minutes of physical activity from baseline to the post-intervention follow-up *F*(1,183) = 9.85, p < 0.01, with a mean difference of approximately 124-minutes per week (p = 0.02), between participants with and without at least 75% program exposure.

### Predictors of program exposure

The IM group were 2.40 times more likely to reach 75% program exposure (95% CI 1.13–5.1), and participants in the IO group were 2.96 times more likely to reach 75% program exposure (95% CI 1.38–6.3) compared to the FACE group. Internet self-efficacy was not a significant predictor (Table 2).

**Table 2:** Odds Ratios for Program Exposure using Baseline Variables for Overall Study Population

**Variable**	**Adjusted OR Model 1^**a, b**^**	**Adjusted OR Model 2^**a, b**^**
**Intervention Group**		
FACE	1.00	1.00
IM	2.40(1.13–5.1)*	1.94 (0.88–4.24)
IO	2.96 (1.38–6.3)*	2.66 (1.21–5.87)*
**Gender**		
Male	1.00	1.00
Female	1.02 (0.46–2.27)	1.09 (0.47–2.52)
**Age**	1.00 (0.98–1.03)	1.01 (0.98–1.03)
**BMI**		
Healthy Weight	1.00	1.00
Overweight	0.73 (0.29–1.82)	0.85 (0.33–2.17)
Obese	0.61 (0.27–1.37)	0.65 (0.28–1.5)
**Internet Self-Efficacy**	0.2 (0.9–1.90)	1.26 (0.86–1.87)
**Physical Activity Self-Efficacy**		1.82 (1.15–2.88)*
**Social Support for Physical Activity**		1.43(0.87–2.35)

*Note*. OR = odds ratio; FACE = face-to-face; IM = internet-mediated; IO = internet-only 1.00 = Reference Category; ^a ^Odds ratios mutually adjusted for all other variables in Model ^b ^Model includes all study participants at baseline; * Statistically significant (p < 0.05)

Results for the overall cohort showed participants with higher PASE were 1.82 times more likely to achieve 75% program exposure (95% CI 1.15–2.88). Participants allocated to the IO group were 2.66 times more likely to be classified as having been exposed to 75% of the program material (95% CI 1.21–5.87) compared to the FACE group (Table 2).

Stratified analysis based upon group allocation (Table 3) showed the FACE group were 79 % less likely to reach 75% program exposure if they were classified as obese (95% CI 0.04–0.96), compared to the IM and IO groups. PASE remained a significant predictor of program exposure in the IM and IO groups (95% CI 1.02–3.3; 1.28–12.25 respectively), and ISE was not a predictor for any of the intervention groups.

**Table 3:** Odds Ratios for Program Exposure using Baseline Variables for the Face-to-Face Group, Internet-Mediated Group, and Internet-Only Group

**Variable**	**Adjusted OR (95% CI) FACE**	**Adjusted OR (95% CI) IM**	**Adjusted OR (95% CI) IO**
**Gender**			
Male	1.00	1.00	1.00
Female	0.18 (0.31–1.03)	2.11 (0.49–9.00)	5.09 (0.96–26.87)
**Age**	0.98 (0.93–1.04)	1.04 (0.99–1.1)	0.99 (0.92–1.06)
**BMI**			
Healthy Weight	1.00	1.00	1.00
Overweight	0.69 (0.12–3.93)	1.24 (0.26–5.87)	2.08 (0.27–16.04)
Obese	0.21 (0.04–0.96)*	1.68 (0.42–6.78)	0.98 (0.17–5.74)
**Internet Self-Efficacy**	1.28 (0.41–1.96)	1.39 (0.67–2.88)	1.84 (0.85–3.99)
**Physical Activity Self-Efficacy**	1.06 (0.95–3.31)	2.14 (1.02–3.3)*	3.97 (1.28–12.25)*
**Social Support for Physical Activity**	0.89 (0.42–2.7)	1.39 (0.59–3.3)	2.31 (0.77–6.95)

*Note*. OR = odds ratio; FACE = face-to-face; IM = internet-mediated; IO = internet-only 1.00 = Reference Category; ^a ^Odds ratios mutually adjusted for all other variables in Model; ^b ^Model includes only participants allocated to the respective intervention group; * Statistically significant (p < 0.05)

## Discussion

Previous studies have acknowledged low participation rates [21,36] and increased drop-out in internet-based interventions [18]. This study found similar declines in weekly program exposure across each intervention group. A significant association between program exposure and participation in physical activity at follow-up (self-report mean minutes of physical activity) was found, suggesting that improved physical activity outcomes were related to being exposed to at least 75% of the Health-*e*Steps program material. In this study, the IO group had higher rates of program exposure and were more likely to have had exposure to at least 75% of the program material compared to the FACE group. Participants with higher PASE in the IM, and IO groups were also more likely to have had at least 75% program exposure. The FACE group participants were 79% less likely to reach at least 75% program exposure if they were classified as obese.

The FACE group showed similar face-to-face attendance rates (exposure) and drop-out, to other physical activity interventions delivered via the same modality [34,37]. However, drop-out in the IO group was lower then the average drop-out rate of 21% reported in a recent internet-based behaviour change meta-analysis [21]. Glasgow et al., [38], reported an overall decrease in website access over time throughout a randomised controlled trial of the Diabetes Network self-management intervention. They reported between 11.4 – 16.7 logins per person during the first three months of the intervention, which decreased to 5.0 – 5.3 per person during the follow-up period (7–10 months). In a study of web-based nutrition counselling for patients at risk of cardiovascular disease, Verheijden [36], reported that only 33% (24/73) of participants used the internet-based program and the study failed to show any increase in outcome measures between the intervention group (internet) and the control group (usual care). They also reported that participants visited the website an average of once over an 8-month period. Another study also reported low exposure to a physical activity website, in which only 46% of participants visited the website at least once, a decline in website access was also observed [20].

One of the strong points associated with the Health-*e*Steps website that may have assisted in user engagement similar to that observed in the FACE group, was the delivery of modules/information sessions on a weekly basis. In this respect, participants could not log on to the website and review the entire website in the one sitting. Further, the website material was built-upon on a week-by-week basis, possibly enticing participants to return to the site the following week. Strategies incorporated into the website such as a "What's New at Health-*e*Steps" section, weekly reminder emails and the numerous interactive features (quizzes, activities, self-appraisal, feedback) may have also enhanced participant engagement in the website. However, this study did not examine specific strategies of engagement, therefore we can only speculate as to why participants returned back to the website.

The Health-*e*Steps program also used incentives to assist in enhancing program exposure across all three intervention groups, however without a comparison group (control with no incentives) this study can not conclude if the incentives had an effect upon program exposure or not. Although the limited observation of participant engagement with the incentive opportunities, suggests that new innovative incentives idea and engagement strategies are required. This may include the examination of different 'types' of incentives, combination of engagement strategies (e.g. email reminders and electronic pedometers that are synchronised with online personal diaries), addition of telephone counselling/support and/or the provision of more automated tailored feedback.

Previous research has also highlighted that poor program outcomes are associated with poor program attendance [13,39]. Tate, Wing and Winett [16], reported greater changes in body weight with increased frequency of website log-on in an internet-based weight loss program. Similarly, this study showed higher program exposure was associated with higher physical activity levels immediately post-intervention. Identifying avenues to increase internet engagement will therefore not only increase program exposure but also lead to an improvement in behavioural outcomes. The internet, as suggested by others [40,41], may be best used as a supplement to face-to-face interventions or standard care. However, we did not show any added advantage for the IM group over the IO, as a result of receiving additional face-to-face program exposure.

A potential avenue for future research is the examination of online chat rooms, support groups, and discussion boards for enhancing treatment effects and participant retention. For the Health-*e*Steps website it was decided not to include a discussion board or online chat room based upon formative evaluation results [26], and the lack of research supporting online chat rooms and discussion boards [17,36]. Further, Harvey-Berino (2002) reported that 'attendance' at an online chat session for the maintenance of weight loss, did not reduce the decline in attendance observed during the intervention. The authors' suggested this is due to participants feeling that they were unable to communicate with other online participants, and the online therapist effectively [40,42]. However, as technology advances and people become more familiar with using various internet components, the use of online support groups, and discussion boards are likely to increase [43]. Therefore, continual investigation of the role and mechanisms that facilitate effective online support (support groups, chat-rooms, discussion boards) is warranted.

To date, few studies have focused upon individual moderators of program effects [44,45], such as the type of person a program is more likely to be effective for [46]. In this context a moderator such as sex or age may strengthen or weaken the relationship between the intervention and the outcome variable [45]. The results of this study, suggest that an internet-based intervention may offer an alternative delivery method to traditional face-to-face delivery for obese participants. This implies that internet-based programs may have a potentially greater outcome effect than traditional face-to-face interventions for this sub-population. This may be partly due to the fact that the internet removes perceived barriers related to weight image and embarrassment as suggested by others [40], and anecdotally expressed by participants in the FACE group. Further, the internet offers advantages over traditional face-to-face programs in terms of autonomy and confidentiality [47,48].

Participants who had higher PASE at baseline were more likely to have been exposed to at least 75% of the program material in the IM and IO groups. Physical activity self-efficacy is commonly reported in the literature as an important mediator [45] across a diverse range of physical activity interventions [49-51]. We did not examine PASE as a mediating variable nor did we examine the relationship between PASE, intention to use the internet, and actual physical activity behaviour. However, the results found may indicate participants who have high PASE may also posses high outcome expectations, and therefore see the potential benefits of using an internet-based behaviour change program.

## Limitations

Interpretation of the findings presented in this study should be mindful of the following limitations. A methodological limitation to this study was that participants allocated to the IM and IO group still received face-to-face contact in Week 1 and Week 12 for data collection. Future studies should therefore, investigate the efficacy of changing behaviour with no face-to-face contact, and use internet-based data collection methods that may also increase the number and reach of the target population [16].

This study compared two types of 'exposure'; face-to-face program exposure (in-person sessions each week for an hour) with internet 'exposure' (logon frequency) in which the access time, day and location was not restricted. These two definitions are inherently different and plausibly reflect differences in the 'dose' of the intervention received. Examination of the amount of 'time' spent on the website (in comparison to a 1-hour session) was unable to be determined. However, the Health-*e*Steps RT was the first study of its kind to compare the efficacy of face-to-face with an internet-based behaviour change program, therefore examining program exposure across delivery modes, as presented here is of interest to researchers. The findings of this study were also based on self-report information and no objective measures of physical activity were used. The sample population were mainly female, living in a rural area of Queensland, Australia, therefore the results are not generaliseable to the population as a whole. Additionally, the study sample was self-selected and may be more motivated to change their behaviour than the general population.

## Conclusion

The findings of this study support the growing body of evidence for the use of the internet in behaviour change research. Weekly exposure showed a similar decline over time, however the internet groups were more likely to have been exposed to at least 75% of the program material. Future research should investigate reasons for internet-based program drop-out and examine strategies for increasing participant exposure. It is also important to explore which target groups or populations that internet-based programs are best suited. Identifying factors that predict drop-out and program exposure is critical for the future development of physical activity internet-based interventions. In this context, we can increase the public health impact of internet-based programs, whilst providing effective alternate delivery modes for increasing physical activity in sedentary populations.

## Competing interests

The author(s) declare that they have no competing interests.

## Authors' contributions

RS participated in the design, development and evaluation of the intervention, performed the statistical analysis, and drafted the manuscript. KM participated in the design and evaluation of the intervention and helped to draft the manuscript. TD helped draft the manuscript. All authors read and approved the final manuscript.
